# A Critical Review of Research Methods Used in: “Use of Risk Assessment Instruments to Predict Violence and Antisocial Behavior in 73 Samples Involving 24,827 People”

**DOI:** 10.5539/gjhs.v5n1p87

**Published:** 2012-11-11

**Authors:** Renee Ann Pistone

**Affiliations:** 1 Rutgers University, New Jersey, United States; 2 California Southern University, California, United States

**Keywords:** predict violence, approach, flaws

## Abstract

Our society requires that experts predict incidences of violence with greater speed and accuracy. We have seen the rise in violence that is random and public. The shootings leave society wondering how could this tragedy have been prevented. Why were the warning signs ignored? This article posits that considering personality traits along with other risk assessments can help make psychologists better predictors of violent behavior.

## 1. Introduction

### 1.1 Clear Statement of Research Aims

One way to evaluate research was to begin with an assessment of whether the researchers have advised the readers about the goals of their research. Here, the authors provided an answer to the question of what was the goal of this research? The goal was to provide readers with a “synthesis of data across a range of accuracy estimates” (Fazel, Singh, Doll, & Grann, 2012) regarding the risk of violence. Why was this research relevant and important? The authors were concerned with time consuming risk assessment tools that were expensive and required specialized training, and “predictive accuracy remained uncertain with false positive decisions,” impacting their effectiveness (Fazel, Singh, Doll, & Grann, 2012). This research related to safety and security concerns and the problem of violence, in our society, as the pressing need to predict and prevent violence, cannot be overestimated.

### 1.2 Research Methods Approach

The article employed a mixture of both qualitative and quantitative research methods. And there was a logical emphasis placed on the use of quantitative methods in the form of statistical analysis. Rather than merely describing the phenomenon and problems with the assessment tools, the article aimed to investigate and to determine the reasons for the false positives. The article may have benefitted from some more detailed descriptions of the problem and more details about the assessment tools, which appeared to be glossed over because the intended audience was assumed to be quite familiar with them. The article did not appear to be written for the layperson but was intended for a clinical specialist concerned with this issue or poor predictions of violence.

### 1.3 Qualitative Data Organized by Table

The descriptions of the assessment tools were broken down into the two main categories: actuarial and structured clinical judgment. Surely, the authors did employ a varied enough usage of these risk assessment tools in their replication studies, making some of their conclusions more reliable. Of note, the article mentioned that PCL-R was not originally intended to be an assessment tool for violence but was a personality assessment. It seemed to me that this tool would be the most useful since it related to the perpetrator’s personality. And, yet, the outcome for this section of **Table 1: Characteristics of nine included risk assessment tools,** was found to be not applicable. If we consider, as Dr. Shapiro pointed out, the perpetrator’s personality proved to be an important, and seemingly overlooked risk factor in domestic violence risk assessments, then not placing some focus on PCL-R may prove to be problematic.

## 2. Nine Assessment Tools

The researchers appeared to utilize an exhaustive list of assessment tools that did not fail to include any known relevant assessment tool. A clinician, however, might be inclined to disagree with that assumption. This section of the article, featuring **Table 1**: employed qualitative data with each type of assessment tool described using headers and variables, such as adult offenders or psychiatric patients. Here, there was less emphasis placed on statistics since the researchers were trying to explain their approach for the readers. The research began the synthesis process by developing tables that highlighted key information that was not limited to statistical information. The tables effectively demonstrated for the readers, at a glance, the instrument type, population, and outcome.

### 2.1 A Relatively Large Sample

The researchers used a large enough sample involving 24,827 people from 13 nations. The scope of the project was extensive and vast making some of the findings seemingly more reliable. The use of samples from different nations appeared consistent with findings that cultural differences should be taken into account in assessments for violence. The article did not specifically mention the reasons why participants from 13 nations were involved but it was inferred. Under the investigation of heterogeneity section, it did mention that evidence was found that “sex, ethnicity, age, type of instrument, temporal design, assessment setting was associated with differences in predictive validity” (Fazal, Singh, Doll, & Grann, 2012).

### 2.2 Predictive Validity and Clinician Liability

Under the clinical implication section, the researchers advised that they were concerned that forms of violence still could not be predicted. It had been mentioned that this research had been studied for 30 years (Fazal, Singh, Doll, & Grann, 2012). And the main problem was that too much pressure and legal determination was placed on the clinician’s ability to predict future violence. This section provided the impetus for the research and the problems with the false positives. Clinicians who did not correctly predict future violence were responsible for confining people who actually had no or a low propensity for violence.

## 3. A Political Issue Rather Than an Ethical Issue

The legal officials, within the 13 nations, still find themselves relying solely on opinions from clinicians when deciding an individual’s fate. The research findings proved that our ability to predict future violence would remain uncertain or not always knowable. Clinicians continued to be faced with high levels of liability, and other severe consequences, stemming from their recommendations. This fact alone would seemingly lead to more recommendations of confinement but it certainly underscored the need for increased certainty.

### 3.1 A Flawed System That Hurts People

In the section entitled, “what this study adds,” this exhaustive study with all of its comparisons from past studies demonstrated that the tools we continued to use, “appear to identify low risk individuals with high levels of accuracy, but have low to moderate positive predictive values, the extent to which these instruments improve clinical outcomes and reduce repeat offending needs further research” (Fazal, Singh, Doll, & Grann, 2012). The researchers concluded in the “what this study adds section,” we were still not in a strong position to make decisions within criminal justice settings, using the tools that we have now (Fazal, Singh, Doll, & Grann, 2012).

### 3.2 Uncertainty Persists

The researchers were concerned with how the process has been politicized because politicians framed the issue as one of public safety. This study did not conduct clinical trials to measure intervention efforts (Fazal, Singh, Doll, & Grann, 2012). It was not entirely clear whether clinical trials would have impacted these research findings. The intervention efforts would have targeted the populations most at risk for future violence. The offenders who have who already committed violent acts appeared to need the most attention from studies. Violent offenders were more likely to repeat based on the 2001 MacArthur Violence Risk Assessment Study. Here, this current body of research from 2012, indicated that it was concerned that it did not fully examine whether predictive validity was different between men and women, but the MacArthur Risk Assessment Study did not indicate that women showed a high propensity for violence, so it may not be worth studying that gender aspect.

### 3.3 Criminal Justice Settings

The criminal justice system and setting continued to place less or no emphasis, on the issue of confinement or continued detainment, as a human rights issue. Yet, individuals continued to be detained or were detained and they may have had no propensity or a low propensity for violence. The system continued to be flawed and detained the seemingly guilty along with the innocent that should not be tolerated. Our criminal justice was structured to do the opposite and has no tolerance for wrongful confinement of an innocent individual. Prosecutors have felt the pains form the so-called exclusionary rule that ensures that an innocent individual would never be confined, and in many cases, the guilty walk free when its stiff provisions were violated by law enforcement.

## Appendix

**Table 1:** Characteristics of nine included risk assessment tools (from “Use of Risk Assessment Instruments to Predict Violence and Antisocial Behavior in 73 Samples Involving 24,827 People”)

Instrument type and name	No of items	Population	Outcome	Current manual
Actuarial				
LSI-R*	54	Adult offenders	Criminal offending	Andrews and Bonta (1995)^32^
PCL-R†	20	Non-specific	Not applicable‡	Hare (2003)^33,34^
SORAG	14	Sexual offenders	Sexual offending	Quinsey et al (2006)^35,36^
Static-99§	10	Sexual offenders	Sexual offending	Harris et al (2003)^37,38^
VRAG	12	Mentally disordered violent offenders	Violent offending	Quinsey et al (2006)^35,36^
Structured clinical judgment				
HCR-20	20	Psychiatric patients	Violent offending	Webster et al (1997)^39,40^
SVR-20	20	Sexual offenders	Sexual offending	Boer et al (1997)^41^
SARA	20	Spousal assaulters	Violent offending	Kropp et al (1999)^42-44^
SAVRY	24	Adolescent offenders	Violent offending	Borum, Bartel, and Forth (2003)^45,46^

* Low and low to moderate risk categories combined to make low risk bin. Moderate to high and high risk categories combined to make high risk bin.

† Psychopathic patients (score >30) considered high risk group, non-psychopathic patients (<30) considered low risk group. PCL-R scores are included in SORAG, VRAG, HCR-20, and SVR-20, and thus the predictive validity of these instruments designed for different outcomes is correlated.

‡ PCL-R was designed as a personality assessment. It started to be used as a risk instrument to predict criminal offending from 1988 onwards.^80^

§ Moderate-low and moderate-high risk categories combined to make moderate risk bin.
